# Multimodal stress reduction and lifestyle modification program for patients with ulcerative colitis: a qualitative study

**DOI:** 10.1186/s12906-021-03478-w

**Published:** 2022-03-08

**Authors:** Christoph Schlee, Christine Uecker, Nina Bauer, Anna K. Koch, Jost Langhorst

**Affiliations:** 1 Department of Internal and Integrative Medicine, Sozialstiftung Bamberg, Buger Str. 80, 96049 Bamberg, Germany; 2 Department of Integrative Medicine, Medical Faculty, University of Duisburg-Essen, Buger Str. 80, 96049 Bamberg, Germany; 3 Department of Sociology, University of Bamberg, Feldkirchenstr. 21, 96052 Bamberg, Germany; 4 Department of Internal and Integrative Medicine, Evang. Kliniken Essen-Mitte, Medical Faculty, University of Duisburg-Essen, Am Deimelsberg 34 a, 45276 Essen, Germany

**Keywords:** Ulcerative colitis, Stress reduction, Lifestyle modification, Qualitative methods, Complementary medicine, Mind-body-medicine

## Abstract

**Background:**

Over 2 million people in Europe are affected by ulcerative colitis, which often severely impacts the quality of life of those concerned. Among other factors, lifestyle and psychosocial factors seem to play an important role in pathogenesis and course of the disease and can be addressed as a complement to pharmacotherapy in comprehensive lifestyle modification programs.

**Methods:**

This qualitative study as part of a mixed methods approach was carried out in the framework of a randomized controlled trial that examined the effect of a comprehensive lifestyle-modification-program (10-week-day clinic program) on quality of life in patients with ulcerative colitis. Qualitative interviews were conducted with 20 out of 47 patients of the intervention group after the program. The aim was to deepen, supplement, and expand the quantitative results of the trial, i.e. to examine individual perceptions of the intervention, including subjective changes and the extent to which elements of the program were integrated into everyday life. Qualitative content analysis techniques utilizing the software MAXQDA were used.

**Results:**

Patients with ulcerative colitis in our sample often experienced multiple negative effects on different levels (physical, psychological, and social) and impaired quality of life because of their disease. They reported generally positively about the program itself, and emphasized perceived positive changes regarding their psychological and physical well-being. The interviews indicated a good implementation of elements learned during the intervention in everyday life.

**Conclusions:**

Through participation in a comprehensive lifestyle modification program in the structure of a day clinic complementary to pharmacotherapy, patients with ulcerative colitis can reduce psychosocial stress and physical symptoms and thereby actively improve their well-being and general quality of life. This patient-centered, holistic approach was rated as useful in countering the complex disease manifestation as well as meeting the individual needs of the patients regarding their disease.

**Trial registration:**

clinicaltrials.gov NCT02721823

## Background

Ulcerative colitis is a chronic inflammatory bowel disease with a prevalence of currently around 2.2 million people in Europe, while the pathogenesis has not been fully understood [1]. Due to the high prevalence, especially in Northern Europe and North America, a connection with the western lifestyle is assumed [2]. Even though there is a genetic risk for the disease, the onset and course are also significantly influenced by environmental and lifestyle factors [1]. For example, the majority of patients report that psychosocial stress has influenced the course of the disease in the past or has even caused a flare [3, 4]. During such a flare, patients often suffer from a severely reduced quality of life. However, patients are restricted in this regard even during inactive phases [5, 6]. In order to reduce psychosocial stress and physical symptoms and improve quality of life, complementary and alternative medicine (CAM) offers a variety of options which, in addition to pharmacotherapy, can have positive effects on patients’ well-being and disease activity. Such therapy options are in great demand for patients with gastrointestinal disorders such as ulcerative colitis [4, 7, 8]. One type of these complementary treatment options are comprehensive lifestyle modification programs which have shown a positive impact on patients’ quality of life [9, 10]. The comprehensive lifestyle modification program of the present study integrates relevant aspects of complementary medicine like mind-body medicine, herbal medicine, nutrition, exercise, and naturopathic self-help strategies within one program. In addition to stabilizing the course of disease, this holistic salutogenetic approach aims to improve patients’ physical and psychological well-being [11]. The quantitative results of this present randomized controlled trial performed by our workgroup showed that a single workshop as well as the 10-week comprehensive lifestyle modification program improved health-related quality of life in patients with inactive ulcerative colitis. Furthermore, the results indicated significant superiority if patients attend at least half of the training sessions of the lifestyle modification program [12]. However, since most complementary treatments like the comprehensive lifestyle modification program are complex in nature, some components of the program have been addressed using a qualitative approach to adequately investigate the program, i.e. to gain further insights, possible explanations and additional understanding from the perspective of the patients participating in the program. Therefore, in addition to the quantitative results, the aim was to deepen, supplement, and extend the randomized trial and its results [13]. Within this mixed methods approach this present interview study has been used to focus on the following questions to enable better understanding of the individual disease and intervention:

- How does the disease (ulcerative colitis) affect the patients’ everyday life?
- How did the patients experience the participation in the program?
- Do patients perceive changes due to the intervention?
- What do patients report about the possibility of integrating elements of the program into their everyday life?

## Methods

### Study design

**Fig. 1:**
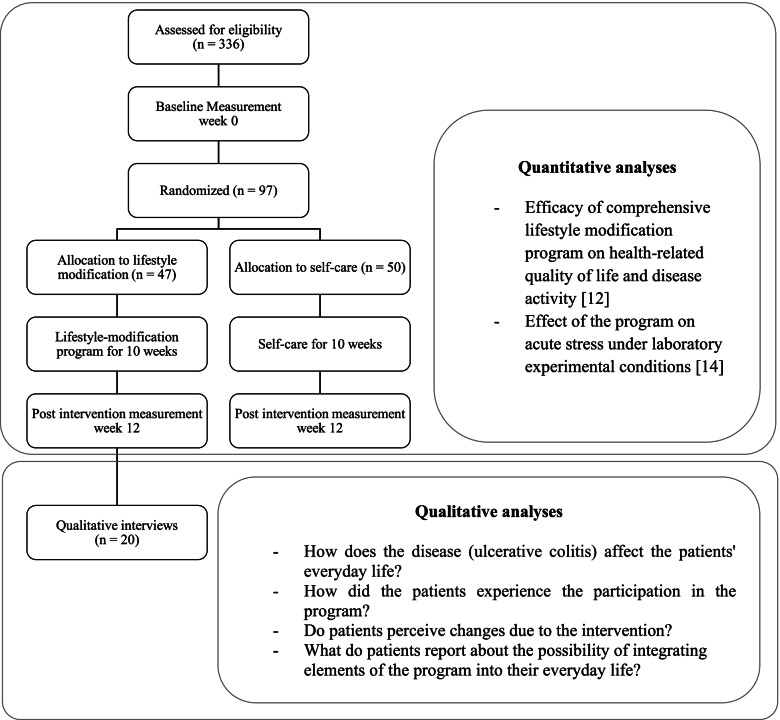
Flow-Chart of mixed methods study design

This qualitative study was conducted as part of a mixed methods approach in the context of a randomized controlled trial at the Kliniken Essen Mitte from 2016 to 2019 that examined the effect of a comprehensive lifestyle-modification-program (10-week-day clinic program) on quality of life in patients with ulcerative colitis [12]. Qualitative interviews were conducted with patients of the intervention group after the program to deepen, supplement, and expand the quantitative results of the trial, i.e. to examine individual perceptions of the intervention, including subjective changes and the extent to which elements of the program were integrated into everyday life. Thus, the qualitative interviews served as a supplementary study to the randomized controlled trial [12, 14] and to answer further in-depth questions (see Fig. 1).

### Comprehensive lifestyle modification program

The comprehensive 60-h lifestyle modification program (see Table 1) consisted of 10 weekly group sessions led by experienced physicians and mind-body instructors. The groups were composed of 8–12 participants who went through the 10 sessions together. The participants received theoretical knowledge and practical training. Topics such as yoga, stress management, mindfulness, herbal medicines, home remedies, communication techniques, meaning of self-awareness and assessment of personal habits and cooking classes were essential in this program. The principal investigator (JL) conducted joint medical rounds. The participants were provided with information material in order to continue at home [12].

**Table 1:**
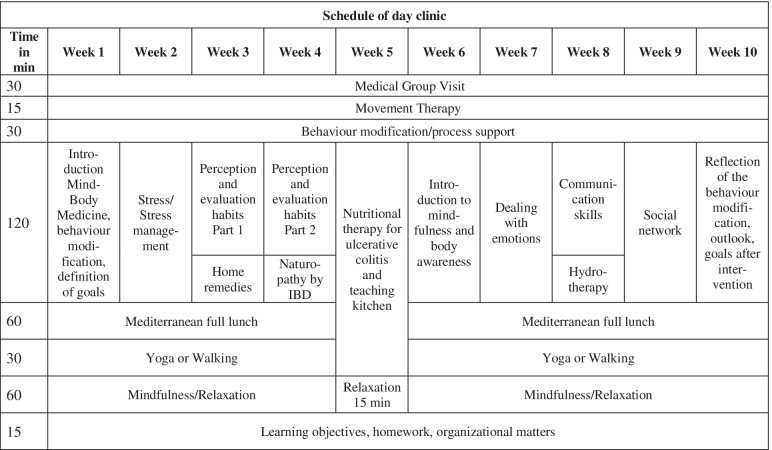
Schedule of day-clinic program

### Participants

A total of 97 patients aged between 18 and 75 years diagnosed with ulcerative colitis who had been in clinical remission for a maximum of 12 months and whose quality of life was impaired were enrolled in the randomized controlled trial, with 47 patients in the intervention and 50 in the control group. Members of the intervention group participated in the program for 10 weeks. The control group did not participate in the program, but received a single two-hour psychoeducational workshop on the topic of naturopathic self-care strategies (e.g. mind-body techniques, herbal medicines and home remedies). In addition, patients were given informational material in the form of a booklet which contains general information about the disease, mind-body medicine and self-help [12]. Patients from the intervention group were invited to voluntarily take part in the semi-structured interviews that took place 2 weeks after termination of treatment. The reason to interview only participants of the program was the specific interest in gaining insights into individual experiences with the program. The purposeful sample for this qualitative study (see Table 2) consisted of 20 patients (*n* = 20) who varied in terms of time since initial diagnosis in years, gender, age in years, and level of education. Thus, it was possible to cover different subjective perspectives and experiences and to identify important common patterns across the interviews [15]. Compared to the entire sample and to the intervention group, the distribution of characteristics in the subsample was similar.

**Table 2:** Main sociodemographic characteristics at baseline of complete sample (randomized controlled trial) and subsample qualitative interviews

	Lifestyle Modification (*n* = 47)	Control (*n* = 50)	Qualitative Interviews (Subsample of Lifestyle Modification group) (*n* = 20)
Age years	50.28 ± 11.90 (18–74)	45.54 ± 12.49 (19–71)	50.80 ± 10.75 (25–71)
Female n (%)	34 (72.3)	35 (70.0)	15 (75.0)
Time since diagnosis in years	18.04 ± 12.00 (2–46)	14.76 ± 10.99 (1–43)	17.00 ± 10.46 (2–42)

Values are expressed as mean ± standard deviation and (min-max)

### Semi-structured interviews

The interviews were conducted at the Kliniken Essen-Mitte at week 12 during the follow-up appointment. To have the possibility to gain specific contents during the interviews, as well as to consider prior theoretical considerations and knowledge, a semi-structured approach using an interview guideline was chosen [16]. Furthermore, this qualitative approach allows gaining previously unexpected insights and enables the participants to bring up their own topics and opinions [17, 18]. The guideline has primarily been developed deductively and contained open questions on the main topics presented in Table 3.

**Table 3:** Main topics and research aims and examples from the interview guideline

Main topics and research aims	Exemplary questions on specific themes from the interview guideline
➢ Experiences with the disease in everyday life and measures to counteract it	Have you had any previous experience with complementary and/or alternative medicine? If so, please tell me about it.
➢ Perception of the program • Satisfaction • Positive and negative aspects • Suggestions for improvement	How did you like the program? What did you find particularly good? What did you find less good?
➢ Integration of elements of the program and impact of participation • Changes in everyday life • Perception of the effectiveness on or change of the disease (e.g. on one’s own well-being) • Acceptance of the disease • Implementation of techniques • Useful aspects and problems/hurdles • Future use of techniques	Do you perceive changes compared to before the program? If so, please describe them. Does the program affect your life? The disease? If so, in what way? How does it manifest itself? How did you integrate contents/measures of the program into your everyday life? Which techniques do you like to use most, which ones less? Why? What was helpful in implementing the program in your everyday life? Do you perceive obstacles? How likely do you think it is that you use the techniques in the future?

The duration of the interviews was approximately 30 min each. Following patients’ consent they were recorded, transcribed verbatim, and anonymized [19]. Some parts have been translated into English for publication purposes. Interview quotations are used here to achieve comprehensibility and transparency as well as to stress the participants’ subjectivity, which also bears on the results and their interpretation [16]. Due to privacy reasons, only the abbreviations P1, P2, P3 etc. are given in the Results section as identification of the interview participants.

### Data analysis

Qualitative content analysis techniques following a content-structuring approach by Kuckartz [20], which is in turn based on the approach by Mayring [21], were used to identify relevant topics and patterns in subjective meanings of the participants [22]. A combination of deductive and inductive elements, i.e. theory-driven and data-driven coding, was chosen. In addition to the determined main and subcategories, further categories were developed interpretatively during the analysis. The development of the categorization system and the application (see Table 4) are based on text interpretation and hermeneutic procedures, which also detect the latent meaning and not only manifest meaning [23]. In addition to a purposeful sampling strategy, i.e., considering a variation of different characteristics of the patients, case comparisons and the identification of patterns in analysis were used to achieve a form of theoretical/content saturation. The interdisciplinary researchers from psychology, sociology, and medicine used the software MAXQDA for the development of codes and coding processes. During the process of analysis, the procedure and the results were discussed and reflected in the research team to ensure transparency and intersubjectivity.

**Table 4:** Main categories of the coding scheme

Main codes	Sub codes
Experiences with the disease	Social level
	Physical level
	Psychological level
Perception of the program	Positive aspects and satisfaction
	Negative aspects and suggestions for improvement
Integration of the program/techniques into daily life	Useful aspects
	Problems/hurdles
	Integration of techniques
Impact of the program/Changes in daily life	Behavioral
	Cognitive
	Emotional
	Social
	Physical

## Results

### The impact of ulcerative colitis on individuals

#### Physical level

On the physical level, patients reported a wide range of symptoms and, resulting from that, severe restrictions in their daily lives. Primarily, gastrointestinal symptoms such as cramp-like pain and diarrhea (sometimes with blood admixtures) *–* in some cases also incontinence *–* were reported. Symptoms were described to be very severe, using terms like “massive” or “extreme” and also extraintestinal manifestations could occur. Moreover, patients reported that medicinal therapies (e.g. treatment with corticosteroids or 5-ASA) can in turn lead to relevant side effects such as skin irritations, hair loss or susceptibility to infection. Exhaustion and fatigue and an overall severely impaired physical condition especially during acute exacerbation were also reported: *“I could not eat anything at all. […] I couldn’t leave (home) at all” (P5)*.

#### Psychological level

Regarding the psychological level, the analyses showed the recurring pattern that patients associated the disease with insecurity, fear, helplessness, and lack of orientation. They narrated that they live in constant fear of a sudden worsening or a flare-up of the disease. Some of them feel left alone by their treating physicians, who sometimes also are indecisive to which therapy to try next. These patients aimed for more guidance or instruction and professional advice. Disappointed by the absence of improvement, some patients turned to complementary and alternative methods of treatment.

Furthermore, several patients reported to feel despondent and dejected, especially during a flare, when a high level of suffering and various restrictions determined their lives. In these times they isolated themselves and even fell into depression: *“I was once in a condition where I really sat in the dark for eight hours and said that I didn’t want to do it anymore” (P4)*.

#### Social level

Beyond that, the interviews show that patients’ generally impaired physical and mental performance was perceived as a burden in professional and private life. The incalculable course of the disease makes long-term planning difficult and sports and leisure activities (e.g. vacation trips) are only possible to a limited extent. A recurrently mentioned aspect was the impact of the disease on family and social life:

*“I can't take part in life anymore. All my friends have turned away because who wants to be out with someone who constantly needs to go to the bathroom or something like that. It's difficult.” (P4*)

The disease requires a lot of understanding and support from colleagues, family and friends. Some reported that they do not feel understood and taken seriously by the “outside world”. Furthermore, the topics “intestine” and “digestion” were reported to be very intimate, tabooed and connected with shame. Communication about it with non-affected people was perceived as very unpleasant by the participants: *“I believe that this problem is rooted in society as one could almost say that colitis is a taboo subject. Because it is very unpleasant to talk about it” (P1).*

Across the interviews, it became clear to what extent the disease determines the patients’ everyday lives. In particular, the problem of imperative stool urgency greatly restricted the patients’ freedom of movement and social life: *“I mean if I have to go to the bathroom, I have to go immediately. And not in a minute but now. That is very unpleasant and that also limits the quality of life very much” (P 5)*.

### Experiences with the program

Regarding the course and structure of the program, the majority of interviewees perceived the teaching of different topics, such as theoretical lessons (e.g. naturopathic self-help strategies, self-medication, nutrition, and phytotherapy) and practical instructions and activities (e.g. exercises, sports, yoga, and relaxation sessions) as a well-balanced variety. A recurring, positively perceived aspect was the frequency of the program (weekly appointment) to intensively address the disease as well as oneself, thus taking the time to focus on one’s body and mind. In this context, there was also positive talk about getting to know the variety of practices and receiving information, emphasizing the voluntary nature of elective practice without pressure. Several patients described that there was an impulse to implement what had been learned. To achieve sustainability, the program provides the necessary tools and is consolidated with material for implementation at home, e.g. CD or printed material for practices.

As already indicated, the majority of patients reported positively on receiving what they considered extensive and useful information (theory and practices; information on implementation, among others nutrition, herbs, teas, bilberry juice, yoga). The scientifically based transfer of knowledge and good preparation and communication were aspects mentioned several times in this context. The focus on the combination of conventional and complementary medicine, and the practical experiences, exercises and strategies for dealing with stress (e.g. relaxation techniques) were emphasized positively across the interviews.

Patients particularly valued that they received additional support in dealing with the disease by means of new approaches and individual consultations, including medical rounds. The program and its positive implementation were supported in particular by the staff, which were characterized as particularly understanding, empathetic and competent.

In addition, the exchange with other persons suffering from the same disease appeared particularly beneficial. Most participants in the sample explicitly mentioned that the possibility of honest exchange with other affected patients enabled them to break the social taboo surrounding the disease, as well as to overcome inhibitions and shame in everyday life. They have benefited from the exchange at the experience level and feel understood: *“I could speak quite freely here, and that’s how I learned a lot from the others, for example about how they themselves actually deal with the disease” (P1)*.

Compared to the various positive aspects of the program, the interviews yielded to a lesser extend complaints or suggestions for improvement. Despite the generally positively perceived balance of individual topics, few participants would have liked a somewhat stronger focus on certain practices or information sessions (e.g. on relaxation, Qi Gong, natural remedies, individual care or everyday life), or less focus on theory. However, in this respect no common pattern across the interviews could be identified. In contrast, common patterns emerged regarding the structure, and sometimes the organization, of the course program: Several participants reported having difficulties integrating the program with employment or other time-consuming commitments, which led to additional strain and stress in everyday life. Finally, five participants of the sample wished to stay in contact with others and suggested further meetings. This confirms the already mentioned positive perceptions of the group meetings.

### Cognitive, behavioral, social, emotional, and physical changes

Overall, the analyses showed that all participants reported changes in at least one of the following domains. At the *cognitive level*, the majority of patients in the sample reported an improvement in their disease acceptance: “*Now I know that it (the disease) will not pass but it will become a companion” (P16).* Some participants mentioned that after the intervention they panicked or felt helpless less often. Knowledge acquisition about the disease and the group setting helped to increase acceptance. In the majority of cases, this improvement went with a change in attitude away from enduring the disease (victim role) to active self-care (e.g. setting boundaries, conducting encouraging self-talk) as well as productive coping with problems (e.g. prioritization of own health over work, usage of learned methods for symptom reduction, completing tasks one after the other). The participants realized and related in the interviews that these changes are lengthy processes that were going to continue. These cognitive changes went along with a higher self-reported ability to concentrate, a reduction of circles of thought, and a feeling of calmness.

*Behavioral changes* were, amongst others a development towards a more conscious, more mindful handling of one’s own body (e.g. finer body sensations, faster perception of body signals and symptoms): *“One has been nudged to pay more attention to the body” (P6)*.

In several cases, an improvement in stress management was reflected in an acceptance of the immutability of stressors (e.g. accepting one’s own mistakes and those of others), increased self-reflection and the active use of strategies to reduce the stress reaction (e.g. breathing exercises), and more time for self-care: “*When I notice that I am not feeling well, I take the time to lie down and relax myself through breathing meditation” (P18).* Breathing exercises were described to induce feelings of calm, relaxation or balance: “*[...] I generally have the feeling that I am more relaxed and calm” (P3)*.

Improving nutritional (e.g. consumption of organic products, reducing meat and sugar consumption, increasing the amount of drinking) as well as exercise behavior (e.g. taking up old or new hobbies, continuing to practice yoga) caused difficulties for some of the participants. They emphasized that the body must first be slowly (re)introduced to movement and that the change in diet led to further stress due to additional shopping and cooking.

The use of naturopathic remedies, such as bilberry juice, psyllium husks etc., could be integrated more easily and were repeatedly described positively in terms of application and effect. In particular, the naturopathic self-help strategies and regular active mindful relaxation were related to symptom improvements: “*In connection with the bilberry juice, [...] I notice that the stool is much more pleasant, […] and I have less pain [...] and that there is no blood left” (P18)*.

*Emotional changes* were reported to be related to a decrease in physical complaints (more relaxed stomach, calmer bowels) as well as increased self-confidence. Some patients were unable to note *physical changes* after the course. However, others perceived improvements related to their bowel movements, bowel noises, pain, and tiredness. In this context, reduced drug (corticosteroid) consumption was reported along with an increase in quality of life:

*“I go to the restaurant and no longer need to look where the toilet is. So I can actually plan the day without a toilet. That wasn't possible for seventeen years. So that's a huge, huge story.” (P20)*

In addition to this possibility of social participation, which was mentioned by some patients and associated with a physical and emotional change, more *social changes* such as fewer conflicts and more frequent conversations with their partner were also reported as positive changes.

### Implementation in everyday life

The participants in this study were encouraged to integrate elements of the multimodal program into their daily routines and practice them outside the clinical program. The interviews revealed that only one participant used one technique; all other interviewees referred to at least two or three techniques associated with different areas of treatment. The techniques mentioned most often and by almost all participants were relaxation (body scan and progressive muscle relaxation) and breathing exercises. Approximately half of the participants also reported implementing the elements of nutrition, phytotherapy, and physical exercise.

#### Obstacles

In the interviews, it was found that lack of time due to family and work commitments is the main obstacle to successful implementation. However, some patients admitted this to be an excuse, the real reason being idleness and convenience. Therefore, the level of implementation seemed to be a question of individual prioritization. Especially during times without acute exacerbation, the level of suffering did not seem to suffice as a motivator.

Sometimes the difficulty to sufficiently reduce the initial stress level was reported as an obstacle to starting relaxation at all. Others reported that at the end of the day they often simply feel too tired for relaxation exercises.

Another referred potential obstacle was that changes in one’s own lifestyle might also have an impact on one’s partner and the whole family life. Sessions of relaxation and exercise need to be integrated into daily family routine and changes in nutrition possibly also affect other family members: If the family does not go along with the changes, different meals have to be prepared. So alterations can make life and daily routines more complex – at least for a while.

#### Helpful aspects

It turned out that some components had a greater chance to become part of the participants’ everyday life than others: A pattern emerged that those that require little effort, time and cost and that were easy to integrate into everyday life and family life had a higher chance of being continued. The interviewees stated that short exercises could be integrated into the daily routine much more easily than exercises that require a relatively long time for preparation and completion. Interviewees aimed to reserve a fixed time in their daily routine which they tried to keep free from disturbances. If an exercise became a “ritual” in everyday life, it was much more likely to be performed on a regular basis.

Patients also described the helpfulness of not having to make special preparations for the performance of the exercise (e.g. no special clothing, room or equipment required) or changes in nutrition and lifestyle. Ideally, the exercises could then be integrated into everyday life with less effort. In addition, changes in diet that do not affect other family members, such as the intake of dietary supplements or phytotherapeutics, could be implemented easily: bilberry juice and psyllium husks are easy to get and use.

It occurred that it could be very helpful if professional and private obligations allow for flexibility in time. Another aspect deemed very important was the support by partner and/or family, i.e. whether close persons show understanding for the exercise and ideally even encourage or participate in it. Nevertheless, most participants were glad if the application had as little impact on their family life as possible.

Particularly supportive, since it was highlighted in almost all interviews, was the material provided during the course. Both the CD with recorded exercises and the information folder supported the implementation at home and were mentioned very often.

In addition, the patients related their appreciation of being guided by experienced and professional therapists and mind-body instructors during the course. The implementation was practiced during the classes, which let the subjects develop some routine and therefore supported internalization of exercises.

It was already mentioned above how important the exchange with other persons with ulcerative colitis was for the participants. This was also seen as a strong motivator for the implementation at home: participants were able to talk about successes and difficulties in the group and learn from the experiences of the others.

Lastly, patients emphasized that the implementation of the learned exercises got reinforced increasingly by way of their positive effect on well-being; for some, the exercises became an indispensable part of their everyday life:

*“When I really got the step to do this (relaxation exercise) regularly, I realized how quickly I became addicted afterwards. […] I was looking forward to that, so that's what changed it completely for me.” (P17)*

To conclude the presentation of the results, Table 5 summarizes the main findings regarding the developed main categories of the coding scheme.

**Table 5:** Summary of main findings

Main codes	Sub codes	Main results
Experiences with the disease	Social level	Patients partly retire from social activities: restrictions due to incalculable course of the disease, difficulties in planning of social and leisure activities, lack of understanding from other people, taboo topic, embarrassing to talk about
	Physical level	Wide range of symptoms: gastrointestinal (pain, diarrhea, incontinence, imperative stool urgency etc.), extraintestinal (e.g. skin), general exhaustion, fatigue
	Psychological level	Impaired quality of life (especially during flare-ups): feeling of insecurity, fear of worsening/upcoming flare-up, helplessness (missing guidance by professionals/treating physicians); despondency/dejection, depressive episodes
Perception of the program	Positive aspects and satisfaction	Provided material (e.g. CD, handouts), combination of theoretical lessons and practical instructions, empathetic and competent staff members, exchange with other persons affected appeared beneficial
	Negative aspects and suggestions for improvement	Integration of program elements into everyday life was partly difficult due to other commitments, participation could lead to additional stress
Integration of the program/techniques into daily life	Useful aspects	Elements requiring little time, preparation and costs are easy to integrate into (family) life, therefore had a higher chance of being continued; reserving a specific timeslot in daily routine increases chance of implementation (ritualized performance)
	Problems/hurdles	Lack of time due to family and work commitments, difficulties in getting started with exercises (e.g. lack of motivation), changes in daily routines have an impact on other family members
	Integration of techniques	Multiple use of techniques: primarily relaxation and breathing exercises, also nutrition, phytotherapy and physical exercise
Impact of the program/changes in daily life	General tendency of perception of positive changes (occurrence and extent case-specific)	
	Behavioral	More mindfulness in handling the own body, improvement of stress management, change in nutrition and exercise behavior, use of self-help strategies
	Cognitive	Improvement in disease acceptance, better knowledge of disease, change in attitude, productive coping with problems
	Emotional	Increased self-confidence in dealing with the disease
	Social	Improved quality of life, increased participation in social activities
	Physical	Improvements related to bowel movements, pain and fatigue (in some cases fewer physical complaints)

## Discussion

This study reveals the following important results: Firstly, patients with ulcerative colitis perceive very extensive, often multiple negative effects of their disease on different levels (physical, psychological, and social). As a consequence, the disease often dominates daily life and impairs quality of life. Secondly, the participants in the multimodal lifestyle modification program generally report very positively about the program itself, especially about contents and structure, e.g. the combination of theoretical and practical parts. Thirdly, they perceive positive changes regarding their psychological and physical well-being. These improvements are evident to varying degrees on an individual basis in the cognitive, behavioral and emotional domains. Some patients experience a reduction in physical complaints as well. Fourthly, the interviews reveal a generally good implementation of intervention elements into everyday life, especially for elements that do not require much time and effort.

The results indicate that the disease is not just limited to the intestine, but also has extraintestinal manifestations as well as far-reaching psychological and social consequences. Impairments to quality of life and mental health [24–27] are evident in respondents’ narratives. Some report a tendency to social withdrawal or depression.

A complex and expansive disease like ulcerative colitis requires a multi-faceted and individualized intervention [28]. Results show that after the intervention patients feel empowered to better manage their disease and play an active role in this process. The program’s holistic nature, by addressing psychological factors in addition to nutrition and exercise, and the voluntary nature of the program’s content, allows for therapy that is tailored to the individual patient and his needs. Psychosocial pathologies can be reduced by a psychosocial intervention. The perceived taboo of the disease in the cases is countered by the informal exchanges, group sessions, and professional rounds and consultations.

While common self-help groups offer an exchange of information and experiences with other affected people [29], this day clinic concept offers a program taught by experienced mind-body instructors and, on top of that, physician supervision and consulting. The interviews reveal this to be a valuable factor, especially for those who report a general feeling of uncertainty and/or a lack of guidance regarding therapy options before the intervention. Approximately 50% of patients with Crohn’s disease or ulcerative colitis use CAM [30], which makes it even more important that physicians provide support and orientation for the wide range of options available [31].

A patient-centered approach considers the individual preferences and CAM experiences of an increasingly critical, demanding group of patients [32, 33] who have many opportunities to obtain medical information, such as on the internet. However, this approach does not replace pharmacotherapy [3, 8, 11], but rather complements it. Positive effects can be achieved, for example, through boosting self-efficacy and relief [8, 11]. Some patients in the sample reported that pharmacotherapy alone has neither proven satisfactory nor sufficient, and thus feel left alone with conventional therapy – they need more than a mere suppression of physical symptoms. This is where potential strengths of this program unfold: it gives patients the opportunity to apply self-help-strategies they learned and experienced to be successful. In this regard, patients can become an active part in the therapy process.

The interviews indicate that participants feel competent and self-confident in implementing what they have learned. In this context, competent guidance and feedback by experienced physicians and mind-body instructors as well as the internalization of practical exercises in a day clinic setting proved to be an important prerequisite for patients for a successful continuation in everyday life. The structure as a day clinic and the incremental witnessing of individual improvements in different domains (physical, psychological, and social) are two drivers, reported by the patients, that help patients overcome their idleness and actively develop an attitude of self-care and mindfulness. Compared to rehabilitation, the day clinic program places stronger emphasis on connecting with everyday routines in order to find suitable applications for each individual patient. The sustainment of motivation and positive effects could be improved in future interventions through “refreshers” and continued exchange after the program, especially since some patients reported that they no longer performed the exercises as consistently when their symptoms improved.

Research shows that perceptions of stress and symptoms are correlated [34]; approximately 70% of patients with ulcerative colitis and Crohn’s disease report that they perceive this link [30]. The present study finds, as indicated by previous research [35, 36], that yoga and breathing strategies can help reduce stress and may be an effective complementary intervention for patients with ulcerative colitis. Patients’ narratives suggest that cognitive and emotional changes interact during a multimodal intervention. However, these changes appeared to be very complex and individual.

Nevertheless, the interviews show that this multimodal program has led to psychosomatic improvements in several cases and, in some cases to improvements on the symptomatic level as well. In addition, the statistical results of the controlled trial [12] indicate the program significantly improved health-related quality of life in patients with ulcerative colitis with mild clinical disease activity and impaired health-related quality of life (Inflammatory Bowel Disease Questionnaire Score < 170 at Baseline). The findings indicate significant superiority if patients attend more than half of the training sessions of the lifestyle modification program (group effect *p* = 0.034 in favor of Lifestyle-modification group). Among these patients who participated in more than 50% of the sessions, 75.68% of the intervention group (51.16% of the control group) experienced a clinically significant improvement of 16 points or more. The perceived improvements due and the positive feedback about the program found in this interview study, in combination with the results from the quantitative part of the randomized trial, indicate that as a complement to conventional treatment, this approach might be beneficial for therapeutic success for a group of ulcerative colitis patients in terms of health-related quality of life and disease progression. However, further studies are needed to confirm this regarding long term effects.

Finally, suggestions for improvement are of great importance for the continuous development of this patient-centered intervention. In addition to small suggestions for content, some patients experienced problems in reconciling the intervention program with work. Having to work on the day of the program or working off absences created additional stress. This could be solved by having the day of participation covered by the public health insurances, i.e. by issuing a sick note. In this way, participation and maximum effectiveness of the program could be made possible for all participants.

### Limitations

Although the study group was quite similar to the typical patient clientele with inflammatory bowel disease making use of CAM [30], generalization of the results is limited due to the qualitative nature of the approach and the focus on patients with ulcerative colitis with mild clinical activity or in remission and decreased quality of life.

During the interviews, social desirability bias or untruthful statements could not be ruled out completely. However, this risk is considered to be low, since the participants had voluntarily participated in the interview part of the study and the interviewers were not involved in the intervention.

Finally, it must be noted that the interviews were conducted shortly after the end of the program. Therefore, the present study does not permit conclusive insights about the medium and long-term effects of the intervention and its implementation in the daily lives of patients.

## Conclusions

This qualitative study complements the results of the randomized controlled trial and indicates that an intervention in the form of a comprehensive lifestyle modification program in the structure of a day clinic as a complement to conventional pharmacotherapy can achieve a variety of benefits for patients with ulcerative colitis. Considering these results of the interview study, together with the findings of the associated randomized controlled trial, this patient-centered and holistic approach proved useful in several cases in countering the complex disease manifestations as well as meeting the individual needs of the patients and can therefore lead, from patients’ point of view, to a higher quality of life (e.g. by reducing psychosocial stress and physical symptoms).

Complementary therapies such as mind-body medicine, herbal medicine, nutrition, exercise and naturopathic self-help strategies can enable patients to actively participate in improving their situation, thereby fostering their perceived self-efficacy. However, further research on the long-term effects of the intervention is needed.

## Data Availability

The datasets used and/or analyzed during the current study are available from the corresponding author J.L. on reasonable request.

## Abbreviation

CAM: Complementary and alternative medicine
